# Evaluation of dose delivery accuracy of Gamma Knife by polymer gel dosimetry

**DOI:** 10.1120/jacmp.v6i3.2110

**Published:** 2005-08-17

**Authors:** Yoichi Watanabe, Tomohide Akimitsu, Yutaka Hirokawa, Rob B. Mooij, G. Mark Perera

**Affiliations:** ^1^ Department of Radiation Oncology Columbia University 622 W168th St. New York New York 10035 U.S.A.; ^2^ Gamma Knife Center Takanobashi Central Hospital 2‐4‐16, Kokutaiji‐cho Naka‐ku Hiroshima; ^3^ Division of Radiation Oncology, Department of Radiology Juntendo University School of Medicine 2‐1‐1 Hongo, Bunkyo‐ku Tokyo 1138421 Japan; ^4^ Department of Radiation Oncology St. Luke 's‐Roosevelt Hospital Center 1000 Tenth Av. New York New York 10019; ^5^ Department of Medical Physics Memorial Sloan‐Kettering Cancer Center 1275 York Ave. New York 10021 U.S.A.

**Keywords:** radiosurgery, 3D dosimetry, polymer gel, MRI, QA

## Abstract

The BANG™ polymer gel dosimeter was used to evaluate 3D absorbed dose distributions in tissue delivered with Gamma Knife stereotactic radiosurgery systems. We compared dose distributions calculated with Leksell GammaPlan (LGP) treatment‐planning software with dose distributions measured with the polymer gel dosimeter for single‐shot irradiations. Head‐sized spherical glass vessels filled with the polymer gel were irradiated with Gamma Knife. The phantoms were scanned with a 1.0T MRI scanner. The Hahn spin‐echo sequence with two echoes was used for the MRI scans. Calibration relations between the spin‐spin relaxation rate and the absorbed dose were obtained by using small cylindrical vials, which were filled with the polymer gel from the same batch as for the spherical phantom. We made voxel‐by‐voxel comparisons of measured and calculated dose distributions for 31×31×31 dose matrix elements. With the 3D dose data we calculated the tumor control probability (TCP) and normal tissue complication probability (NTCP) for a simple model. For the maximum dose of 100 Gy, the mean and one standard deviation of differences between the measured and the calculated doses were the following: –0.38±4.63 Gy,1.49±2.77 Gy, and –1.03±4.18 Gy for 8‐mm, 14‐mm, and 18‐mm collimators, respectively. Tumor control probability values for measurements were smaller than the calculations by 0% to 7%, whereas NTCP values were larger by 7% to 24% for four of six experiments.

PACS numbers: 87.53.‐j, 87.53.Dq, 87.53.Ly

## I. INTRODUCTION

Many investigators are actively studying polymer gel dosimeters as a dosimetric tool that provides true 3D dose distributions in a tissue‐equivalent medium.^(^ ^1^ ^)^ There are several studies in which polymer gel dosimeters were used with Gamma Knife systems (Elekta AB, Stockholm, Sweden).^(^ ^2^ ^–^ ^7^ ^)^ With the polymer gel dosimeter we can evaluate the mechanical and geometrical accuracy of shot placement of Gamma Knife units. Furthermore, we can verify the dose calculation accuracy of the Leksell GammaPlan (LGP) treatment‐planning software. Since the experimental procedure with the polymer gel dosimeter closely simulates patient treatment, the measurements can identify the errors involved with the entire treatment procedure.

The dose delivery accuracy of Gamma Knife systems was investigated by measuring dose distributions. Some studies examined the shot placement accuracy of Gamma Knife units by measuring the distance between the location of the dose maximum and the mechanical center.^(^ ^8^ ^–^ ^10^ ^)^ Comparison of 1D line profiles and 2D dose distributions between measurements and LGP calculations was undertaken by some investigators.^(^ ^2^ ^–^ ^7^ ^)^ There are other studies for which Monte Carlo simulation methods were used to evaluate the LGP calculations.^(^ ^11^ ^–^ ^13^ ^)^ The results of previous studies show that the distance between the measured center of shots and the mechanical center (or the unit center point) is 1 mm or smaller. The distances between the corresponding isodose curves between measurements and LGP calculations vary from 1 mm to 3 mm when comparisons are made for 1D dose profiles or 2D dose distributions. Our results, which are not presented in this paper but have been published in part elsewhere,^(^ ^14^ ^,^ ^15^ ^)^ are consistent with the observations by other investigators concerning 1D or 2D comparisons.

An ultimate goal of our project is the clinical application of the polymer gel dosimeter for routine quality assurance of Gamma Knife systems. To our knowledge, there is no publication that shows a true 3D comparison between measurements and LGP calculations. In this work, we compare 3D dose distributions measured with a polymer gel dosimeter with LGP‐calculated dose distributions. However, we present the results for single‐shot experiments only. More complicated cases using multiple shots will be presented in the future.

## II. MATERIALS AND METHODS

We used the BANG™ polymer gel from MGS Research Inc. (Guilford, CT).^(^ ^16^ ^,^ ^17^ ^)^ The BANG™ polymer gel records 3D dose distributions produced by radiation beams. Permanent images of the dose distributions are made visible in the transparent gel by radiation‐induced polymerization. Quantitative dose distributions can be obtained using MRI, CT, ultrasound, or optical CT.^(^ ^18^ ^–^ ^21^ ^)^

For this study, the dose distributions in the BANG™ polymer gel were evaluated with a 1.0T Gyroscan MRI scanner (Philips Medical Systems, Best, the Netherlands). We used a spin‐echo pulse sequence. Scanning parameters were the following: TR=2000 ms,TE=20 ms and 100 ms, slice thickness=2 mm or 3 mm, field of view =24 cm×24 cm, and matrix size=256×256.

**Figure 1:**
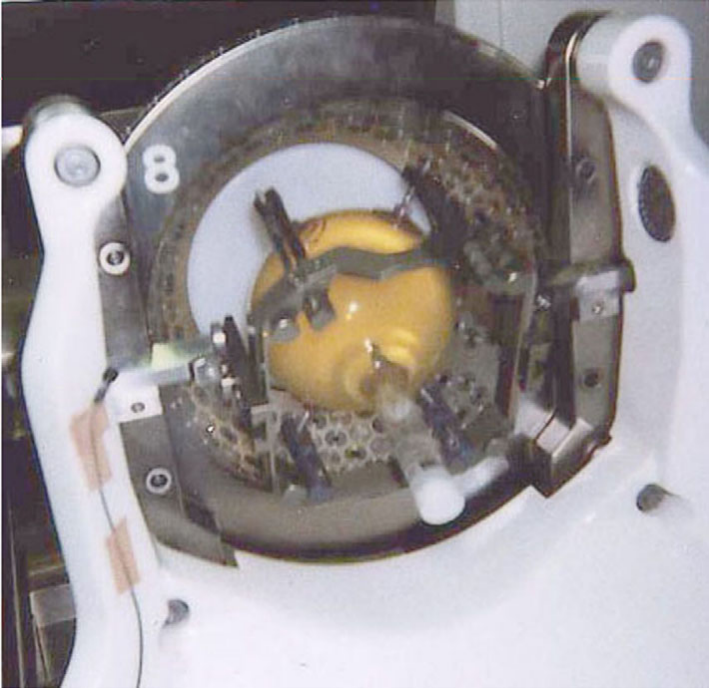
The phantom placed in the Gamma Knife unit for irradiation

A 16‐cm diameter spherical Pyrex flask was filled with BANG™ polymer gel. Recent studies of polymer gel dosimetry for Gamma Knife used small polymer gel‐filled containers embedded in a head‐shaped solid phantom.^(^ ^5^ ^,^ ^7^ ^)^ These setups introduce unknown effects of material inhomogeneity on dose distributions and may lead to large measurement uncertainties. Using a large polymer gel phantom enables us to create a uniform medium the size of a human head. Figure 1 shows the phantom placed in a Gamma Knife unit for irradiation.

The experimental procedure was as follows: First, the phantom was mounted in the Leksell stereotactic frame. Second, the depth to the unit center point (UCP) was measured in the same way as with a patient. Third, the phantom was irradiated. Shots were placed at UCP for all experiments. We gave 25 Gy to the 50% isodose level, leading to the maximum dose of 50 Gy at the UCP. After the irradiation, the phantom was scanned with an MRI scanner. Note that the experimental procedure closely simulates patient treatment except the MRI scanning and the irradiation were in a reversed order. The experiments were repeated for three collimator size helmets: 8 mm, 14 mm, and 18 mm. A total of six experiments were carried out by varying collimator size, γ angle, and MRI slice thickness. See Table 1 for a summary of the measurement setup. For each experiment, polymer gel from a different production batch was used, resulting in slightly different dose response characteristics.

**Table 1:** Setup parameters for irradiation experiments

Experiment ID number	Collimator (mm)	γ angle	MRI slice thickness (mm)
1	8	90	3
*2*	8	90	2
3	8	110	2
4	14	90	3
5	14	110	2
6	18	90	3

We obtained a calibration relationship between absorbed dose and spin‐spin relaxation rate (R2). We used small cylindrical vials filled with polymer gel from the same batch as the main phantom. The vials were placed in water and irradiated to known doses with parallel opposed beams. The calibration vials were attached to the main phantom for MRI scans. A simultaneous MR scanning of the calibration vials and the main phantom ensures the identical MR imaging condition. We computed R2 values by assuming an exponential decay of the MR signal.^(^ ^4^ ^)^ Then the R2 images were converted to dose images by applying the calibration equation.

The LGP saves calculated dose distribution data in a 3D matrix form. The matrix size is 31×31×31. The UCP, where shots were placed, corresponded to the center of the dose matrix. The grid sizes used for calculations were 1.2 mm, 2.0 mm, and 2.5 mm for 8‐mm, 14‐mm, and 18‐mm collimator experiments, respectively.

Measured dose was represented as a 256×256×60 matrix. The voxel size was 0.94mm×0.94mm×2mm or 3 mm. Measured dose and LGP dose matrixes were coregistered manually by using the fiducial markers in the Leksell localization box. The measured dose matrixes were converted to a smaller matrix, whose elements are the doses at the points corresponding to the spatial points of the LGP dose matrix. For this conversion we applied a 3D linear interpolation method as implemented in the MATLAB software (MathWorks, Inc., Natick, MA).

During the course of this study, we found that a direct application of calibration data to convert the R2 image data to the dose distributions in larger phantoms was inaccurate. Similar results have been observed in the past.^(^ ^22^ ^)^ The calibration data had to be modified by rescaling the measured dose and changing the background dose value.

We compared measured and calculated doses at the same points in space. Using measured and calculated dose matrixes with the same size (or 31×31×31), we calculated dose differences at all points in the dose matrixes (or 29 791 spatial points). The dose difference was defined as a subtraction of the calculated dose from the measured dose. Note that for the comparison, the measured dose was normalized so that the maximum dose was 100 Gy. The dose difference values (or 29 791 data points) were grouped according to the dose difference and the dose level of the LGP calculation at the point. Then, with a 5‐Gy interval, the mean and the standard deviation of the dose difference were computed. The results are presented in a diagram called the “dose‐dependent dose‐difference (D4) diagram.” Three‐dimensional dose comparisons were commonly made by plotting two isodose distributions, dose differences, or the gamma values on planes.^(^ ^22^ ^,^ ^23^ ^)^ More quantitative analyses were done with a differential dose‐volume histogram, in which the percent volume is plotted as a function of dose difference.^(^ ^24^ ^)^ The D4 diagram adds the dose dependence to the differential dose‐volume histogram and aids identifying global differences between two dose distributions in 3D. Local differences, however, can be visualized only by displaying two dose distributions on a plane.

Another method for comparing dose distributions in 3D is to calculate the tumor control probability (TCP) and the normal tissue complication probability (NTCP). We applied the Goitein model^(^ ^25^ ^)^ and the Lyman model^(^ ^26^ ^,^ ^27^ ^)^ for TCP and NTCP calculations, respectively. The TCP and NTCP calculations were done for model geometry. We assumed a spherical tumor at the center of the dose matrix. The tumor diameters approximately corresponded to the diameter of the sphere covered by the 50% isodose surface. Note that 50% is a typical prescription isodose line for Gamma Knife radiosurgery. The outside of the tumor was assumed to be normal brain tissue. For LGP calculations the maximum dose of 100 Gy was delivered to the UCP. As mentioned earlier, 50 Gy was given to the UCP for experiments. For the analyses, therefore, measured doses were normalized to 100 Gy at the UCP. The model parameters for TCP calculations were the dose required for 50% probability of tumor control, TCD50, and a measure of the slope of the dose response curve, $\gamma_{\text{gamma}}$. We used TCD50=50 Gy and $\gamma_{50}=0.5$.^(^ ^28^ ^)^ The parameters for NTCP calculations were the dose that results in a 50% probability of complication over 5 years, TD50/5, and two parameters, *n* and *m.* We set TD50/5=60 Gy,n=0.25, and m=0.15.^(^ ^29^ ^)^ Note that only the relative values for the maximum dose, TCD50, and TD50/5 affect the TCP and NTCP values.

## III. RESULTS

**Figure 2:**
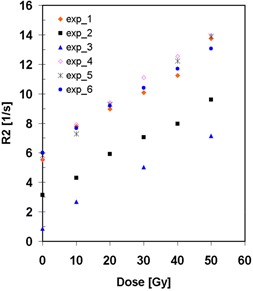
Calibration data: spin‐spin relaxation rate R2 versus absorbed dose

Figure 2 shows calibration data of R2 versus absorbed dose for six independent experiments. The calibration relations are close to each other for four of six experiments. The R2 values for experiments 2 and 3 were much smaller than for the other four cases. This might be caused by differences in ambient conditions, such as the temperature during the transportation and storage of the polymer gel. Note that the polymer gels were exported from the United States to Japan. Error bars are not shown in the figure; in fact, one standard deviations with the measurement data were smaller than 4%. The calibration data were fitted with a linear or a quadratic equation, whichever is more appropriate, to calculate the doses for given R2 values.

**Figure 3:**
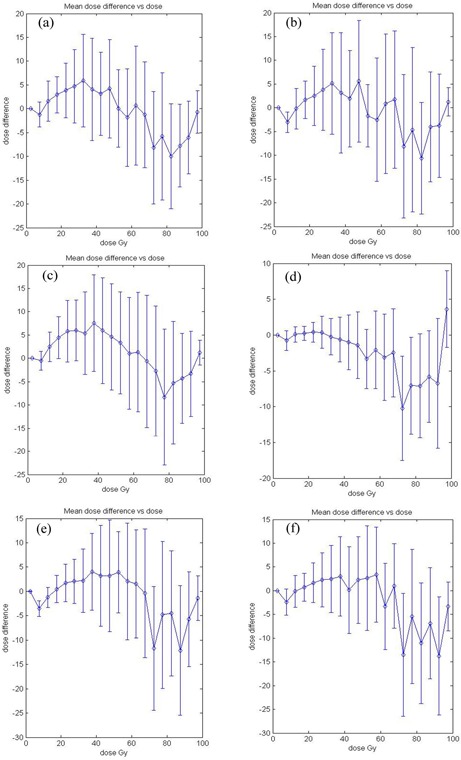
Dose‐dependent dose‐difference diagram (D4 diagram). The diamond symbols indicate the mean dose difference. The error bars are for one standard deviation. Dose difference is calculated by subtracting measured dose from LGP‐calculated dose. The maximum dose is normalized to 100 Gy or 100% for both measured and calculated dose distributions. (a) Experiment 1: 8 mm collimator; (b) experiment 2: 8 mm collimator; (c) experiment 3: 8 mm collimator; (d) experiment 4: 14 mm collimator; (e) experiment 5: 14 mm collimator; (f) experiment 6: 18 mm collimator.

Figure 3 shows the D4 diagram for six experiments. Note that the dose difference is calculated by subtracting the measured dose from the LGP‐calculated dose, and the unit is the gray. Since the maximum dose is normalized to 100 Gy, percent and gray are interchangeable for representing the dose difference. Mean dose differences (diamonds) and standard deviation errors (bars) are displayed. (Figures 3a), (b), and (c) show the results for the 8‐mm collimator helmet. The mean dose differences range from +2% to +8% at 40 Gy and from –5% to –10% at 80 Gy. (Figures 3d) and (e) are the results for the 14‐mm collimator helmet. (Figure 3e) shows a trend similar to the 8‐mm collimator results, whereas (Fig. 3d) shows good agreement for doses lower than 70 Gy. The 18‐mm collimator helmet result given in (Fig. 3f) shows that the measured doses are lower by 4% to 14% than the calculations in the 70 Gy to 100 Gy dose range. All results except experiment 4 ((Fig. 3d) exhibit a common pattern: the measured doses in the 20 Gy to 70 Gy range are larger than the LGP‐calculated doses, and the measured doses are smaller than the calculations in the 70 Gy to 90 Gy range.

**Figure 4:**
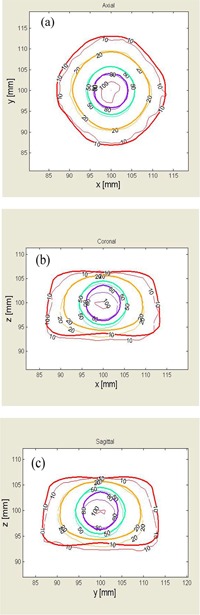
Dose distributions plotted on orthogonal planes for experiment 3. Thin lines are isodose lines of the measurement, and thick lines are calculated by LGP. (a) Axial plane; (b) coronal plane; (c) sagittal plane.

In Fig. 4, the dose distributions of experiment 3 (8‐mm collimator) are plotted on three orthogonal planes, which pass through the UCP. The distributions on the axial plane in (Fig. 4a) indicate that the 10%, 20%, 50%, and 80% isodose lines agree to within a 1‐mm distance between the measurements (thin lines) and the LPG calculations (thick lines). However, the distributions on the coronal plane, Fig. 4 (b), and on the sagittal plane, (Fig. 4c), show clear differences between the measurements and the calculations. The 10%, 20%, and 50% isodose lines of the measurements are wider in the z‐direction (or the axial direction) than those of the LGP calculations. The 80% isodose line by the measurement is narrower in the *z*‐direction than the calculation. These results are consistent with the dose differences observed in (Fig. 3c).

We obtained differential dose‐volume histograms by taking statistics of the number of data points as a function of dose difference. The results are summarized in Table 2. The mean dose difference is less than 2.4%, and the standard deviations vary from 2% to 4.8%. The mean and one standard deviation of differences between the measured and the calculated doses were the following: –0.38±4.63 Gy,1.49±2.77 Gy, and –1.03±4.18 Gy for 8‐mm, 14‐mm, and 18‐mm collimators, respectively. Note, however, that the minimum and maximum dose differences are large, indicating the wide spread of the dose difference.

**Table 2:** Means, standard deviations, minimum, and maximum of dose differences for four collimators. All values are in percent.

Experiment ID number	Mean	Standard deviation	Minimum	Maximum
1	–0.15	4.59	–28.5	33.1
2	–1.76	4.80	–37.1	32.9
3	0.76	4.51	–35.6	34.2
4	–0.55	2.00	–25.8	34.2
5	–2.44	3.53	–33.3	30.7
6	–1.03	4.18	–31.4	32.2

Table 3 presents the TCP and NTCP values for three collimator size helmets. Tumor control probability values calculated with measured dose distributions are smaller than LGP‐calculated dose distributions by 0% to 7%. Larger differences between the calculations and the measurements, that is, 7% to 24%, are seen with the NTCP values because of 5% higher measured doses in the 20 Gy to 70 Gy dose range, except for experiments 4 and 6, for which the measured doses in the medium dose range were not much larger than the calculated doses.

**Table 3:** Radiobiological comparison. LGP indicates Leksell GammaPlan calculations, and BANG means the measured dose.

Experiment	Tumor Diameter^a^	TCP		NTCP	
ID number	(mm)	LGP	BANG	LGP	BANG
1	10.0	68.0	64.2	7.5	8.6
2	10.0	68.0	63.3	7.5	8.0
3	10.0	68.0	65.1	7.5	9.3
4	16.0	77.3	75.7	9.3	8.7
5	16.0	77.3	74.3	9.3	10.2
6	25.0	57.9	58.0	6.1	5.9

a Tumor diameter corresponds to the size of 50% isodose surface.

## IV. DISCUSSION

In this study we calculated dose differences. In regions with a large dose gradient, however, the spatial distance between corresponding isodose surfaces is often considered a better parameter for comparison.^(^ ^30^ ^)^ Since the spatial resolution of calculations as well as measurements is limited by the grid size, there are dose uncertainties due to the finite grid size. We estimated dose gradient at the 50% dose level using the dose profiles measured with GafChromic™ films (ISP, Wayne, NJ). We calculated the dose uncertainties by multiplying half the grid size by the dose gradient. The results are shown in Table 4. Note that the estimated uncertainties are almost the largest possible values because the dose gradient at the 50% dose is the steepest. For any collimator size, we can expect uncertainties of 9 Gy to 10 Gy (or 9% to 10% if the dose is normalized to 100%). Therefore, most of the dose differences we observed in this study correspond to the distances between isodose surfaces that are smaller than half the grid size. The data given in Table 4 can be also used to estimate the distances between isodose surfaces of measured and calculated dose distributions for a given dose difference. For example, a 9% dose difference between measurements and calculations is equivalent to a 1 mm displacement of 50% isodose surfaces for 14‐mm collimator shots.

**Table 4:** Estimated dose uncertainties due to the finite grid size for dose calculations and measurements

Collimator (mm)	Half of grid size (mm)	Dose gradient (Gy/mm)	Dose uncertainty (Gy)
8	0.6	18.1	10.9
14	1.0	9.1	9.1
18	1.25	7.3	9.1

For this study we performed manual coregistration of measured and calculated dose distributions using the fiducial markers in the Leksell localization box. The validity of this procedure is partially supported by our previous study, which showed the mechanical placement accuracy of single shot within 2 pixels.^(^ ^4^ ^)^ A more rigorous approach for image coregistration including rotational transformation will computationally match the fiducial markers instead of the current visual trial‐and‐error approach.

## V. CONCLUSIONS

This work is the initial attempt to experimentally evaluate the 3D absorbed dose distributions calculated by LGP. The point‐by‐point dose comparison in 3D showed systematic global differences, which were common to three collimator sizes: 8 mm, 14 mm, and 18 mm. However, the dose differences between LGP calculations and measurements were small when the measurement uncertainty was considered. Tumor control probability values calculated with measured doses agreed with those with LGP‐calculated doses fairly well, whereas NTCP values calculated with measured doses were larger due to larger measured doses in the medium dose range of 20% to 70%.

Applications of polymer gel dosimetry to Gamma Knife, in particular, to single‐shot experiments, are the first step toward applications of this technique to more complicated dose delivery cases such as multiple‐shot Gamma Knife treatment plans, small field dosimetry common to stereotactic radiotherapy, and intensity‐modulated radiation therapy. The methods and evaluation tools developed for this study will be invaluable for future studies.
